# Risk Perceptions of Antibiotic Usage and Resistance: A Cross-Sectional Survey of Poultry Farmers in Kwara State, Nigeria

**DOI:** 10.3390/antibiotics9070378

**Published:** 2020-07-04

**Authors:** Ahmad I. Al-Mustapha, Victoria O. Adetunji, Annamari Heikinheimo

**Affiliations:** 1 Department of Veterinary Services, Kwara State Ministry of Agriculture and Rural Development, Ilorin 240213, Kwara State, Nigeria; 2 Department of Veterinary Public Health and Preventive Medicine, Faculty of Veterinary Medicine, University of Ibadan, Ibadan 200284, Oyo State, Nigeria; vo.adetunji@mail.ui.edu.ng; 3 Department of Food Hygiene and Environmental Health, Faculty of Veterinary Medicine, University of Helsinki, 00710 Helsinki, Finland; annamari.heikinheimo@helsinki.fi; 4 Finnish Food Authority, 00790 Seinäjoki, Finland

**Keywords:** antibiotic usage, antibiotic resistance, poultry, KAP, Kwara, Nigeria

## Abstract

Overwhelming empirical evidence has highlighted the contribution of indiscriminate antibiotic usage (ABU) in food animals to the overall burden of antibiotic resistance (ABR) in humans, thus making antibiotic use the main selective pressure driving antibiotic resistance. The social and behavioral perspective on antibiotic use and resistance in poultry is limited. Our study therefore aimed at obtaining information on antibiotic usage, awareness of ABR, and the attitude and perceptions towards prudent antibiotic usage and ABR. A cross-sectional survey using a structured questionnaire was conducted in 125 poultry farms in Kwara state in December 2019. Most farmers (69.6%, *n* = 87/125) were aware of ABR and had satisfactory knowledge about ABR with a mean knowledge score of 3.2 ± 1.5. Age (older farmers; OR: 1.1, 95% CI: 1.0, 1.2) and gender (male respondents, OR: 8.5, 95% CI: 3.0, 23.9; *p* < 0.01) were more likely to have satisfactory knowledge of ABR. Tertiary education was significantly associated with ABR awareness (OR: 4.7; 95% CI: 0.1, 0.7; *p* = 0.007) and the ABR knowledge level (OR: 7.8; 95% CI: 3.3, 18.7; *p* < 0.01). Higher flock size was significantly associated with a satisfactory knowledge of ABR (OR: 9.5; 95% CI: 3.8, 23.6; *p* < 0.01). Most of the poultry farmers (68%) had positive attitudes towards prudent antibiotic use with a mean score of 2.7 ± 0.9. On the contrary, only 32.8% of poultry farmers had a desirable perception of ABR with a mean perception score of 4.9 ± 1.1. The ABR knowledge level was significantly associated with the perceptions of farmers (*p* < 0.05) but not their attitudes toward ABU and ABR (*p* = 0.083). There was evidence of unprescribed use of antibiotics in poultry and a failure to observe antibiotic withdrawal periods. These constitute a risk of exposure to unacceptable levels of drug residues from poultry products and an increased risk of ABR. Improving education and communication on antibiotic stewardship programs are crucial to prevent the looming antibiotic threat.

## 1. Background

The illicit and uncontrolled access to and use of antibiotics in humans and animals is one of the major drivers of antibiotic resistance (ABR). It is a global health threat that is estimated to cause approximately 10 million deaths and over USD 100 trillion by 2050 if no global actions were established [1,2]. It is estimated that the greatest impact of ABR will be in Sub-Saharan Africa and Asia due to the disproportionately high infectious disease burden, overwhelmed health care systems of most countries, poor livelihoods and living conditions, and poor healthcare infrastructures. Globally, ABR has gained global attention due to the increasing incidence of multidrug-resistant (MDR) organisms causing treatment failures, antibiotic residues in food, and the public health risks it poses [3]. There is an increase in the incidence and dissemination of MDR organisms in humans, health facilities, animals, foods, and the environment [4,5,6,7,8,9].

In Nigeria, the misuse of antibiotics, proliferation of unlicensed drug stores, sub-therapeutic use of antibiotics in food animals for prophylaxis and growth promotion, poor ABR awareness, and lack of stewardship programs have further frustrated efforts at controlling ABR in the human and animal health sectors [10]. In Nigeria today, most antibiotics are available as over-the-counter (OTC) drugs. According to the antibiotic use and resistance situational report in Nigeria, the overall outpatient antibiotic prescription rate was 49.1% [11]. A point prevalence study showed 80% of all hospitalized patients were on an antibiotic course [12]. In a 2015 global survey by the world health organization (WHO), Nigeria was the country with the highest number of respondents who reported having obtained antibiotics from a stall or hawker [13].

With a population of over 200 million people, there is extensive economic pressure on the poultry industry as a vital source of animal protein for the teeming population of Nigeria. The poultry industry in Nigeria has 180 million birds and produces 650,000 metric tonnes of egg and 300,000 metric tonnes of poultry meat annually [14]. Globally, there are increasing empirical evidence and epidemiological studies highlighting the contribution of indiscriminate antibiotic usage (ABU) and ABR in animals to the overall burden of ABR in human [15,16,17]. This interconnectedness of ABU and ABR in animals and human health requires a multi-sectoral one health approach [18].

There is a paucity of data on ABU and ABR from a social and behavioral change perspective which is essential to achieve attitudinal change necessary to control the imminent threat of ABR. Hence, improving the public awareness and knowledge on prudent use of antibiotics will play a significant role in reducing the illicit consumption of antibiotics, the main driver of ABR especially in the animal health sector in Nigeria. As such, we aimed to assess the knowledge, attitude, and perceptions of poultry farmers regarding ABU and ABR.

This paper is part of a broader project on improved animal health and disease demographics in Nigeria.

## 2. Materials and Methods

### 2.1. Ethical Considerations

The Kwara State Ministry of Agriculture and Natural Resources, Ilorin, Nigeria (reference number: VKW/714/1/103) approved this study. Participation was anonymous and voluntary. Informed consent was sought from the respondents and participants could withdraw from the survey at any time in line with stipulations of the World Medical Association Declaration of Helsinki Ethical principles [19].

### 2.2. Study Design, Study Participants, and Sample Size

This study was conducted in December 2019 as a cross-sectional survey of poultry farmers in Kwara State. A comprehensive list of poultry farms was obtained from the Ministry of Agriculture and the Poultry Association of Nigeria (*n* = 197 farms). The targeted respondents were farm owners or managers. To calculate the sample size, we hypothesized that at a 95% confidence level, the assumed prevalence of antibiotic use was 50% of all farms. The total sample size was 131 farms. So, a random sampling of 131 farms was done to evaluate antibiotic usage and the farmer’s perception of ABR.

### 2.3. Questionnaire Design

A structured questionnaire was designed to conduct this study. It was designed by a team of public health veterinarians as part of the baseline survey of the epidemiology unit of the State Ministry of Agriculture (Supplementary file 1). The questionnaire was pre-validated by two independent reviewers, and a pilot study was conducted with 10 respondents. The responses from the pre-test were not included in the analyzed data. The questionnaire consisted of 4 parts: a) Demography of respondents, b) Antibiotic usage c) Knowledge of antibiotics and ABR d) Attitude and perceptions towards ABR. Some of the questionnaires were administered to farmers in their farms (*n* = 80) while others (*n* = 45) were administered to farmers in feed milling plants when they came to purchase poultry feeds.

### 2.4. Numeric Scoring System

Each graded question was allotted 1 point (Table 1). A total score for each respondent was computed. The mean score was used as the cut-off for a satisfactory outcome (Knowledge, attitude, or perception). Hence, respondents with mean scores that were greater than the mean scores for knowledge (3.2 ± 1.5), attitude (2.8 ± 0.9), and perception (4.9 ± 1.1) were deemed to be satisfactory responses and vice versa (Table 2).

### 2.5. Data Analysis

Data were summarized using Microsoft Excel 2016 and analyzed using Minitab v.19.1.1. Descriptive statistics (frequency and proportions) were used to summarize the obtained data. To assess the knowledge, attitude, and perception levels of the poultry farmers, a numeric scoring system was used (Table 1), and outcome variables—knowledge, attitude, and perception—were computed. These outcome variables were further categorized as binary (satisfactory or unsatisfactory) based on the cut-off (mean scores) marks. Chi-square test was used to test for association between independent variables (demographics) and outcome variables (knowledge, attitude, and perception) at a 95% confidence interval with significant variables (*p* < 0.05) subjected to a logistic regression model.

## 3. Results

### 3.1. Respondent Demographics

The questionnaire was administered to 131 poultry farmers. However, only 125 responses were received as 6 farmers did not consent to participate in the study. Of these, female respondents accounted for most (56.8%, *n* = 71) of the responses. Most poultry farmers (72%, *n* = 90) had tertiary education and 63 (50.4%) of all farmers employed 1–2 workers (Table 3).

### 3.2. Antibiotic Usage in Poultry

Most of the farms (89%, *n* = 111) had layers and 87% (*n* = 109) of all farms had less than 1000 birds (small scale farmers). Similarly, 95.2% (*n* = 119) of all farms have completed their vaccination schedule against endemic poultry diseases. The majority of farmers (83.2%, *n* = 104) used antibiotics in the last 4 weeks but only one farm (0.8%) took samples for laboratory testing before the administration of antibiotics. During this survey, gentamicin based (68.8%, *n* = 86), sulfonamide (44%, *n* = 55), and quinolone-based antibiotics (30.4%, *n* = 38) were the most frequently administered antibiotics in poultry. Most of the farmers (92%, *n* = 115) purchased antibiotics from licensed drug stores. The majority of farmers (56%, *n* = 70) purchased antibiotics based on their previous experiences (Table 4).

### 3.3. Knowledge, Attitude, and Practices towards Antibiotic Resistance in Kwara State

The majority of the poultry farmers (69.6%; *n* = 87) were aware of antibiotic resistance. The mean knowledge score was 3.2 ± 1.5. Using the mean score as the cut-off, most of the poultry farmers (87; 69.6%) have satisfactory knowledge about ABR. Most farmers (72%, *n* = 90) knew that bacteria in poultry could become resistant to drugs but only 49 farmers (39.2%) agreed that ABR could make treatment difficult in birds. On the contrary, 39 (31.2%) of the farmers were unaware that ABR pathogens in birds can affect man. Additionally, 43 farmers (34.4%) were unaware that antibiotics cannot be used to treat viral, fungal, or parasitic infections in birds (Table S1). The age and the level of education of farmers were significantly associated with increased ABR awareness (*p* < 0.05). Poultry farmers with secondary education were 4.7 × (95% CI: 0.1, 0.7; *p* = 0.007) more likely to be aware of ABR than those with tertiary education (Table 5). Similarly, the age, gender, level of education of farmers, and their flock size were significantly associated with a satisfactory knowledge of ABR (*p* < 0.05) (Table 5, Table S2). Male farmers were 8.5 *×* (95% CI: 3.0, 23.9; *p* < 0.01) more likely to have satisfactory knowledge. An increase in flock size was significantly associated with a satisfactory knowledge of ABR (OR: 9.5; 95% CI: 3.8, 23.6; *p* < 0.01) (Table 5). Respondents that have commercial poultry farms (but not full-term farmers) were 2.0 × (95% CI: 0.9, 4.6; *p* = 0.111) more likely to have satisfactory knowledge than full-term poultry farmers (Table S2).

Most of the poultry farmers (68%, *n* = 85) had positive attitudes towards prudent antibiotic use with a mean score of 2.8 ± 0.9. Most farmers (88%, *n* = 110) did not believe that there was excessive antibiotic use in poultry. Only (48.8%, *n* = 61) farmers got their antibiotic prescription from a vet. While most farmers (89.6%, *n* = 112) claim to observe the withdrawal period of antibiotics as stipulated on each antibiotic sachet or vial, none of them discarded the eggs in the course of antibiotic therapy (Table S3). The farmer’s age was significantly associated with a positive attitude towards prudent antibiotic usage (*p* < 0.05) (Table S4).

On the contrary, only 41 farmers (32.8%) had a desirable perception of ABR with a mean perception score of 4.9 ± 1.2. Most farmers (85.6%, *n* = 107) did not believe that ABR is a major health threat that needs to be addressed in Nigeria. Similarly, 84 farmers (67.2%) did not believe that farmers must reduce the use of antibiotics in birds. Only 59 farmers (47.2%) felt that only a certified veterinarian should be allowed to prescribe antibiotics. Some farmers (48%, *n* = 60) of the farmers believe that proper routine vaccinations could reduce dependence on antibiotics. Most of the farmers (72%, *n* = 90) thought that the threat of ABR only affects farms that use antibiotics. While only 56 farmers (44.8%) thought there is nothing that they can do to reduce the emergence and transmission of MDR bacteria, most farmers (81.6%, *n* = 102) believed that frequent hand washing is important for poultry farmers after attending to their birds (Table S5). Farmer’s education was significantly associated with a good perception of ABR (OR: 0.1; 95% CI: 0.1, 0.3; *p* < 0.01) (Table 5, Table S4).

The ABR knowledge level was significantly associated with the perceptions of farmers (*p* < 0.05) but not their attitudes toward ABU and ABR (*p* = 0.083) (Table 6). The flock size of the farmers was significantly associated with awareness rates as well as knowledge and perception (*p* < 0.05) but not with the attitude of poultry farmers (*p* = 0.468) (Table S2; Table S4). Secondary education was the major predictor of higher ABR awareness and a good perception of ABR (Table 5).

## 4. Discussion

Frequent and sub-therapeutic doses of antibiotics create the ideal selective pressure for the emergence of resistant micro-organisms. The excessive use (or misuse) of antibiotics in animal production has severe consequences for public health and the environment [6,9]. MDR organisms have been isolated in poultry, poultry environment, and in poultry workers and this poses serious public health threats especially in LMICs like Nigeria [20,21,22,23]. These MDR bacteria can be transmitted to humans via the food chain, the environment, water bodies, or by close contact with these animals [6,24,25].

In this study, the majority of farmers (83.2%) used antibiotics in their poultry during the last four weeks but only one (0.8%) farm took samples for laboratory testing before the administration of the antibiotics. For economic reasons, small-scale poultry farmers did not consult veterinarians before the administration of antibiotics and the majority of farmers (56%) purchased antibiotics based on their previous experiences (Table 3). In Nigeria, a cocktail of antibiotics with multivitamins and mineral elements is common. Gentamicin, sulfonamide, and quinolone-based antibiotics were the most frequently administered antibiotics in these birds. This is similar to reports by Adebowale et al., [26] and Ogunleye et al., [27] where gentamicin, tetracycline, quinolones, and sulfonamides were the most frequently used antibiotics in poultry in Ogun state. Several other studies have also estimated the ABU and ABR in livestock, and gentamicin and tetracycline were antibiotics most reported by farmers (Table 7). Their popularity amongst farmers might be because they are very cheap and readily available [28]. Although 95.2% of all farms had completed their vaccination schedule against endemic poultry diseases in Nigeria, only some (48%) knew that vaccinations could prevent the occurrence of disease and this will reduce antibiotic consumption. The misuse, abuse, and resistance to quinolones (ciprofloxacin) in animals are particularly worrisome because ciprofloxacin is on the essential medicines list for humans [29]. Surprisingly, over the last two months (8 weeks), no farmer reported the use of any of the banned antibiotics in animals such as furazolidone and chloramphenicol.

While the majority of poultry farmers (69.6%) were aware of ABR, there were obvious gaps in their knowledge of ABR. Some poultry farmers (34.4%) thought antibiotics can be used to treat viral, parasitic, and fungal diseases. This might be due to the use of antibiotics to treat secondary bacterial infections associated with viral diseases such as Newcastle disease or fowl pox. Although most farmers were familiar with ABR as a term, they do not know what it means and the implications for human health. This is further evidenced by the fact that most farmers knew that bacteria in birds could become resistant to antibiotics but were unaware that these resistant bacteria could make treatment difficult in birds. Older farmers had significantly higher ABR awareness rates, knowledge levels, and attitudes than younger respondents. This might be due to hands-on experience acquired over the years. Farmers with secondary education were more aware and had better attitudes and perceptions of ABR than those with tertiary education. However, those with tertiary education had better knowledge of ABR (Table S4). Male farmers were more likely to have a better knowledge of ABR than females. Therefore, there is a need to improve ABR awareness among female farmers. This can be achieved by collaborations with women in agriculture groups throughout Nigeria. Farmers with higher flock sizes were more aware and had better knowledge of ABR. However, for economic reasons and production pressure, these variables do not influence the attitude of poultry farmers towards prudent antibiotic use.

The attitude of most poultry farmers towards ABR (68%) was positive with a mean score of 2.8 ± 0.9. However, through enhanced behavioral changes communications, the attitude of poultry farmers needs to be improved with an emphasis on the observance of withdrawal periods for antibiotics, and the public health impact it has on human health. There is a plethora of studies that reported the non-adherence to antibiotics withdrawal periods as the major cause of antibiotic drug residues in foods of animal origins [3,31,32,33,34,35,36]. As previously reported by Adebowale et al., [27] and Geidam et al., [37], some poultry farmers (38.4%) in this study stopped antibiotic therapy when they noticed improvements in their birds.

With a mean of 4.9 ± 1.1, only 32.8% of poultry farmers had a satisfactory perception of ABR Most poultry farmers neither believed they practice excessive antibiotic use nor perceived the imminent threat of ABR. Following this, most farmers (67.2%) did not see the need to reduce antibiotic use in birds. The majority of poultry farmers did not believe that ABR is a major health threat, hence the national Action plan for antimicrobial resistance (NAP-AMR) should be focused on raising awareness of ABR in these farmers. This is even more important with the increasing number of poultry farmers in Nigeria. Farmers should also be educated that mechanical transmission of drug-resistant microorganisms is possible in farms that do not use antibiotics. Farmers could introduce MDR-organisms into their farms from feed mills, through feed sacks, transport vehicles, and farm workers. To contribute to the fight against ABR and preserve the efficacy of drugs in humans and animals, farmers should practice good management practices, administer essential poultry vaccinations, tighten the biosecurity measures on their farms, and make use of prebiotics and probiotics.

The major limitation of the study was the possibility of desirability bias during questionnaire administration. Additionally, underlying contextual and cultural factors such as years of poultry experience, previous history of disease occurrence, and age of farmer could have impacted the responses of poultry farmers. For future studies, it is essential to establish a positive correlation between farms that used certain antibiotics and an increase in drug resistance pathogens associated with those antibiotics administered. This is important to infer the impact of ABU on the development of resistance in birds.

We would like to promote the concept of antibiotic-free birds among consumers of poultry products. It is essential to assess and quantify antibiotic usage in other livestock production systems such as in aquaculture and dairy farms.

## 5. Conclusion

This study reports the extensive use of unprescribed essential antibiotics in poultry. The majority of antibiotics were received as OTC drugs and the withdrawal period of antibiotics were not followed. Farmers knew about ABR but were unaware of their harm for their animals and the potentials for human transmission. Therefore, interventions such as the full implementation of the NAP-AMR, antibiotic stewardship programs, and behavioral change communications to livestock farmers should be instituted to prevent the looming antibiotic threat. Furthermore, regulations restricting the un-prescribed sale of antibiotics should be enforced. This public health threat can only be solved by multi-sectoral collaborations using the one-health approach [38]. Furthermore, a functional national ABR surveillance program in the livestock sector is long overdue.

## Figures and Tables

**Table 1:** The survey instrument and the numeric scoring system used to evaluate the knowledge, attitude, and perceptions of poultry farmers in Kwara state.

Instrument	Grading System
Knowledge (*n* = 6)	
Awareness of ABR	No = 0; Yes = 1
Can antibiotics be used to treat viral, fungal, or parasitic infections in birds?	No = 0; Yes = 1
Can ABR pathogens in birds affect man?	No = 0; Yes = 1
Can poultry be resistant to drugs?	No = 0; Yes = 1
Does ABR make treatment difficult in birds?	I don’t know, No = 0; Yes = 1
Can ABR pathogens be transferred between humans?	No = 0; Yes = 1
Attitude (*n* = 5)	
Do you believe there is excessive antibiotic usage in birds?	No = 0; Yes = 1
Do you stop treatment when your birds have shown improvements?	No = 0; Yes = 1
Did you get an antibiotic prescription from a vet?	No = 0; Yes = 1
Do you observe the withdrawal period of antibiotics?	No = 0; Yes = 1
Do you discard eggs during antibiotic therapy?	No = 0; Yes = 1
Perception (*n* = 8)	
Is ABR a major problem in Nigeria?	No = 0; Yes = 1
Only vets should be allowed to prescribe antibiotics	1-3 = 0; 4-5= 1
Farmers must reduce antibiotic use	1-3 = 0; 4-5 = 1
Proper vaccination will reduce dependence on antibiotics	1-3 = 0; 4-5 = 1
Antibiotic resistance can only affect farms that use antibiotics	No = 0; Yes = 1
Antibiotics should only be prescribed when needed	1-3 = 0; 4-5 = 1
There is nothing I can do to stop antibiotic resistance	1-3 = 0; 4-5 = 1
Is hand hygiene important for poultry farmers?	No = 0; Yes = 1

**Table 2:** Description of scores obtained by respondents for knowledge, attitude, and perception towards antibiotic resistance (ABR) (*n* = 125).

Outcome Variables	Maximum Obtainable Scores	Scores Received by Respondents		Mean ± SD	Satisfactory *n* (%)	Unsatisfactory *n* (%)
		Minimum score	Maximum score			
Knowledge	6	0	6	3.16 ± 1.47	87 (69.6)	38 (30.4)
Attitude	5	0	4	2.75 ± 0.89	85 (68)	40 (32)
Perception	8	3	7	4.95 ± 1.12	41 (32.8)	84 (67.2)

**Table 3:** Demographic structure of respondents (*n* = 125).

Variables	No. of Respondents (%)
Gender	
Female	71 (56.8)
Male	54 (43.2)
Age	
20–30	26 (20.8)
30–40	18 (14.4)
40–49	39 (31.2)
50–59	29 (23.2)
60–69	13 (10.4)
Level of Education	
Secondary education	35 (28)
Tertiary education	90 (72)
No of workers on the farm	
1	31 (24.8)
2	32 (25.6)
3	20 (16)
4	13 (10.4)
5	9 (7.2)
6	14 (11.2)
7	4 (3.2)
9	2 (1.6)

**Table 4:** Descriptive statistics on poultry flocks and antibiotic usage in selected poultry farms in Kwara state (*n* = 125).

Variables	No. of Respondents (%)
Type of bird	
Broilers	12 (9.6)
Cockerel	2 (1.6)
Layers	111 (88.8)
Population of birds	
<500	37 (29.6)
500–1000	72 (57.6)
>1000	16 (12.8)
Ever sampled birds for lab testing?	
No	124 (99.2)
Yes	1 (0.8)
Vaccination status	
Complete vaccination schedule	119 (95.2)
Incomplete vaccination schedule	6 (4.8)
Was vaccination done by a vet?	
NO	104 (83.2)
YES	21 (16.8)
When last did you administer antibiotics to your birds?	
<4 weeks	104 (83.2)
>4 weeks	21 (16.8)
Which class of antibiotics did you use?	
Gentamicin based	86 (68.8)
Quinolones based	38 (30.4)
Sulphadimidine based	55 (44)
Oxytetracycline based	40 (32)
Sources of antibiotics	
Drug peddlers	7 (5.60)
Licensed store	115 (92)
Other sources	3 (2.40)
Do you purchase antibiotics based on other farmers’ experiences?	
No	101 (80.8)
Yes	24 (19.2)
Do you purchase antibiotics based on your own previous experiences?	
No	55 (44)
Yes	70 (56)

**Table 5:** Predictors of ABR awareness, satisfactory knowledge, and perceptions of ABR among poultry farmers in Kwara state.

Predictors	Variables	Univariate Analysis		Multivariate Analysis	
		OR (95% CI)	*p-*Value	OR (95% CI)	*p-*Value
Awareness of ABR	Secondary education	4.7 (1.5, 14.5)	0.007	2.2 (0.1, 5.3)	<0.01
Knowledge of ABR	Tertiary education	7.8 (3.3, 18.7)	<0.01	2.2 (0.7, 6.5)	<0.01
	Male Gender	8.5 (3.0, 23.9)	<0.01	4.2 (1.2, 14.8)	0.027
	Flock size between 500–1000	9.5 (3.8, 23.6)	<0.01	4.5 (1.6, 13.2)	0.021

**Table 6:** Association of knowledge level and the attitude and perceptions of poultry farmers on ABR.

Attitude						
**Knowledge**		**Good (%)**	**Poor (%)**	**χ2**	**DF**	***p-*** **Value**
	Satisfactory	55 (64.7)	32 (80)	3.01	1	0.083
	Unsatisfactory	30 (35.3)	8 (20)			
	**Perception**					
	Satisfactory	16 (39)	71 (84.5)	26.96	1	<0.01
	Unsatisfactory	25 (61)	13 (15.5)			

OR—Odds ratio; 95% CI—95% confidence interval.

**Table 7:** Comparison of antibiotics usage across Nigeria and Africa.

Location	Animals Involved	Production System	Prevalence of Antibiotics Use	Most Frequently Used Antibiotics	Reference
Kwara State, Nigeria	Poultry	Intensive	83.2%	Gentamicin based (68.8%)	This study
Ogun State, Nigeria	Poultry	Intensive	-	Gentamicin based (76.7%)	[26]
Niger State, Nigeria	Cattle	Extensive	-	Tetracyclines (96.6%).	[20]
Ethiopia	Ruminants	Extensive	43.9%	Tetracyclines (36.4%)	[30]
Ghana, Kenya, Tanzania, Zambia, and Zimbabwe (Multi-country study)	Poultry Ruminant	Intensive Extensive	-	Tetracyclines (70%)	[6]

## Supplementary Materials

The following are available online at https://www.mdpi.com/2079-6382/9/7/378/s1, Supplementary file 1. Survey instrument used in this study. Supplementary file 2. Table S1. Frequency and proportion of respondents’ knowledge of ABR (*n* = 125). Table S2. Analysis of demographic characteristics as factors influencing knowledge, attitude, and perception levels of poultry farmers in Kwara state. Table S3. Frequency and proportion of respondents’ attitude towards prudent antibiotic usage in poultry (*n* = 125). Table S4 Analysis of demographic characteristics as factors influencing knowledge, attitude, and perception levels of poultry farmers in Kwara state. Table S5. Frequency and proportion of respondents’ perceptions on ABU and ABR in poultry (*n* = 125).
