# Supplementary material for: Risk Perceptions of Antibiotic Usage and Resistance: A Cross-Sectional Survey of Poultry Farmers in Kwara State, Nigeria
Source: Antibiotics (Basel). 2020 Jul 4;9(7):378. doi: 10.3390/antibiotics9070378 (PMC7400114; doi:10.3390/antibiotics9070378)
Supplement: Supplementary file 1 [file antibiotics-09-00378-s001.pdf]

Supplementary Data

Supplementary file 1. Survey instrument used in this study.

Populatn of birds	Category		
200	<500	100	<500
190	<500	200	<500
500	500 - 1000	300	<500
500	500 - 1000	400	<500
850	500 - 1000	500	500 - 1000
444	<500	600	500 - 1000
250	<500	700	500 - 1000
389	<500	800	500 - 1000
440	<500	900	500 - 1000
500	500 - 1000	1000	>1000
500	500 - 1000	2000	>1000
450	<500	3000	>1000
480	<500	4000	>1000
179	<500	5000	>1000
480	<500		
480	<500		
470	<500		
350	<500		
500	500 - 1000		
210	<500		
400	<500		
500	500 - 1000		
500	500 - 1000		
495	<500		
450	<500		
500	500 - 1000		
500	500 - 1000		
350	<500		
800	500 - 1000		
450	<500		
500	500 - 1000		
500	500 - 1000		
300	<500		
800	500 - 1000		
500	500 - 1000		
900	500 - 1000		
500	500 - 1000		
450	<500		
440	<500		
550	500 - 1000		
470	<500		
470	<500		
200	<500		
999	500 - 1000		
450	<500		
450	<500		
800	500 - 1000		
350	<500		
400	<500		
450	<500		
400	<500		
400	<500		

450	<500
850	500 - 1000
999	500 - 1000
600	500 - 1000
440	<500
600	500 - 1000
750	500 - 1000
2500	>1000
850	500 - 1000
800	500 - 1000
800	500 - 1000
600	500 - 1000
600	500 - 1000
650	500 - 1000
750	500 - 1000
470	<500
890	500 - 1000
900	500 - 1000
400	<500
999	500 - 1000
999	500 - 1000
999	500 - 1000
999	500 - 1000
999	500 - 1000
999	500 - 1000
999	500 - 1000
2700	>1000
999	500 - 1000
3500	>1000
999	500 - 1000
500	500 - 1000
2500	>1000
700	500 - 1000
550	500 - 1000
650	500 - 1000
600	500 - 1000
900	500 - 1000
999	500 - 1000
999	500 - 1000
3300	>1000
800	500 - 1000
800	500 - 1000
999	500 - 1000
400	<500
900	500 - 1000
900	500 - 1000
1000	>1000
980	500 - 1000
3000	>1000
2500	>1000
5000	>1000
800	500 - 1000
990	500 - 1000
520	500 - 1000
500	500 - 1000
600	500 - 1000
509	500 - 1000
600	500 - 1000

1000	>1000
2500	>1000
900	500 - 1000
988	500 - 1000
550	500 - 1000
2000	>1000
2200	>1000
750	500 - 1000
750	500 - 1000
1000	>1000
1000	>1000
900	500 - 1000
550	500 - 1000
2000	>1000
670	500 - 1000

Supplementary file 2.

Table S1. Frequency and proportion of respondents' knowledge of ABR (n = 125).

Awareness of ABR	No. of respondents (%)
No	38 (30.4)
Yes	87 (69.6)
Can antibiotics be used to treat viral, fungal, or parasitic infections in birds?	
No	82 (65.6)
Yes	43 (34.4)
Can ABR pathogens in birds affect man?	
No	86 (68.8)
Yes	39 (31.2)
Can poultry be resistant to drugs?	
No	35 (28)
Yes	90 (72)
Does ABR make treatment difficult in birds?	
Don't know	62 (49.6)
No	14 (11.2)
Yes	49 (39.2)

Table S2. Analysis of demographic characteristics as factors influencing knowledge, attitude, and perception levels of poultry farmers in Kwara state.

Outcome variable	Variable	Referent		OR (95% CI)	*p-value*	OR (95% CI)	*p-value*
			Univariate analysis			Multivariate analysis	
Awareness of ABR	Age	18 years	19 years and above	1.0 (1.0, 1.1)	0.009	1.1 (1.0, 1.2)	< 0.01
	Level of Education	Secondary	Tertiary	0.2 (0.07, 0.7)	0.007	0.05 (0.01, 0.2)	< 0.01
	Gender	Female	Male	0.8 (0.4, 1.7)	0.534	-	-
	Population of birds	100-499	500-1000	1.6 (0.7, 3.7)	0.494	-	
			>1000	1.8 (0.5, 6.8)			
Knowledge level of ABR	Age	18 years	19 years and above	1.1 (1.1, 1.2)	< 0.01	1.1 (1.0, 1.2)	0.033
	Level of Education	Secondary	Tertiary	7.8 (3.3, 18.7)	< 0.01	2.2 (0.7, 6.5)	< 0.01
	Gender	Female	Male	8.5 (3.0, 23.9)	< 0.01	4.2 (1.2, 14.8)	0.027
	Population of birds	100-499	500-1000	9.5 (3.8, 23.6)	< 0.01	4.5 (1.6, 13.2)	0.021
			>1000	1.2E+07 (0.0, *)		1.0E+06 (0.0, 2.1E+297)	
	Occupation	Farmers	Other professions	2.0 (0.9, 4.6)	0.111	-	
	No. of workers	1	2 and above	1.1 (0.9, 1.4)	0.310	-	

Table S3. Frequency and proportion of respondents' attitude towards prudent antibiotic usage in poultry (n=125).

Do you believe there is excessive antibiotic usage in birds?	No. of respondents (%)
No	110 (88)
Yes	15 (12)
Do you stop treatment when your birds have shown improvements?	
No	77 (61.6)
Yes	48 (38.4)
Did you get antibiotic prescription from a vet?	
No	64 (51.2)
Yes	61 (48.8)
Do you observe withdrawal period of antibiotics?	
No	13 (10.4)
Yes	112 (89.6)
Do you discard eggs during antibiotic therapy?	
No	125 (100)
Yes	0 (0)

Table S4. Analysis of demographic characteristics as factors influencing knowledge, attitude, and perception levels of poultry farmers in Kwara state.

Outcome variable	Variable	Referent		OR (95% CI)	*p-value*	OR (95% CI)	*p-value*
			Univariate analysis			Multivariate analysis	
Attitude towards ABR	Age	18 years	19 years and above	1.0 (0.9, 1.0)	0.038	-	-
	Level of Education	Secondary	Tertiary	0.5 (0.2, 1.3)	0.175	-	-
	Gender	Female	Male	1.3 (0.3, 1.6)	0.506	-	-
	Occupation	Farmers	Other professions	0.6 (0.3, 1.3)	0.194	-	-
	No. of workers	1	2 and above	1.1 (0.9, 1.4)	0.267	-	-
	Population of birds	100-499	500-1000	0.7 (0.3, 1.8)	0.705	-	-
			>1000	0.6 (0.2, 2.1)			
Perception of ABR	Age	18 years	19 years and above	0.9 (0.8, 0.9)	< 0.01	0.9 (0.9, 1.0)	< 0.01
	Level of Education	Secondary	Tertiary	0.1 (0.1, 0.3)	< 0.01	0.3 (0.1, 0.8)	0.015
	Population of birds	100-499	500 -1000	0.5 (0.2, 1.2)	0.062	-	-
			>1000	0.2 (0.0, 0.8)			
	Gender	Female	Male	1.3 (0.4, 1.6)	0.506	-	-
	Occupation	Farmers	Other professions	0.5 (0.2, 1.2)	0.109	-	-
	No. of workers	1	2 and above	0.9 (0.8, 1.1)	0.535	-	-

Table S5. Frequency and proportion of respondents' perceptions on ABU and ABRin poultry (n=125).

Is ABR a major problem in Nigeria?	No. of respondents (%)
No	107 (85.6)
Yes	18 (14.4)
Only vets should be allowed to prescribe antibiotics	
1 (Strongly disagree)	17 (13.6)
2 (Disagree)	20 (16)
3 (Neither agree nor disagree)	29 (23.2)
4 (Agree)	40 (32)
5 (Strongly agree)	19 (15.2)
Farmers must reduce antibiotic use	
1 (Strongly disagree)	64 (51.2)
2 (Disagree)	20 (16)
3 (Neither agree nor disagree)	9 (7.2)
4 (Agree)	21 (16.8)
5 (Strongly agree)	11 (8.8)
Proper vaccination will reduce dependence on antibiotics	
1 (Strongly disagree)	29 (23.2)
2 (Disagree)	31 (24.8)
3 (Neither agree nor disagree)	5 (4)
4 (Agree)	5 (4)
5 (Strongly agree)	55 (44)
Antibiotic resistance can only affect farms that use antibiotics	
No	90 (72)
Yes	35 (28)
Antibiotics should only be prescribed when needed	
1 (Strongly disagree)	26 (20.8)
2 (Disagree)	6 (4.8)
3 (Neither agree nor disagree)	23 (18.4)
4 (Agree)	10 (8)
5 (Strongly agree)	60 (48)
There is nothing I can do to stop antibiotic resistance	
1 (Strongly disagree)	0 (0)
2 (Disagree)	7 (5.6)
3 (Neither agree nor disagree)	62 (49.6)
4 (Agree)	17 (13.6)
5 (Strongly agree)	39 (31.2)
Is hand hygiene important for poultry farmers?	
Don't know	23 (18.4)
No	0 (0)
Yes	102 (81.6)
